# 2024 MCBK North American chapter meeting—Lightning talk and demonstration abstracts

**DOI:** 10.1002/lrh2.10479

**Published:** 2025-01-03

**POSTERS**

1. Boddapati, Saketh OMOP‐2‐OPMI: Ontologization of OMOP Common Data Model Using the Ontology of Precision Medicine and Investigation for Standardized Electronic Health Data Representation and Integration.
2. Bray, Bruce Interoperability Standards to Support Computable Biomedical Knowledge in Learning Health Systems.
3. Flynn, Allen The Evolution of the Knowledge Object Model Enhances the FAIRness of CBK.
4. Gauthreaux, Nicole Real‐World Measurement Experience to Mobilize Computable Biomedical Knowledge for Patient‐Centered Clinical Decision Support.
5. Goyal, Pawan From Information to Intelligence: Driving Data Transformation in Emergency Medicine.
6. Kahanda, Indika Explainable Automated Inconsistency Detection in Biomedical and Health Literature.
7. Landis‐Lewis, Zach A Computable Knowledge Base for Precision Feedback.
8. Limaye, Siddharth Representing and Inferring from Medical Knowledge in a Typed Database.
9. Misro, Aswini Bridging the Trust Divide: How Medical AI Outperforms Yet Battles to Win Public Confidence.
10. Osheroff, Jerome Identifying Care Gaps to Drive Clinical Quality Improvement: Interoperable, Computable Biomedical Knowledge‐Enabled Approaches are Urgently Needed.
11. Santos, Henrique Eliciting Survey Knowledge with Semantic Data Dictionaries.
12. Swain, Deborah Open Educational Resources (OER) on MCBK.
13. Swain, Deborah Using KM to Manage AI Bias in Healthcare.
14. Tian, Yujia Towards Reconstruction of the Human Phenotype Ontology Psychiatry Section by Learning from Traditional Psychiatric Diagnostic Infrastructures.

**DEMONSTRATIONS**

1. Bierman, Arlene Interoperable EHR Data for Comprehensive Care Planning, Evidence Generation, and Implementation Among People Living with Multiple Chronic Conditions.
2. Huff, Stanley The LOINC Ontology: A LOINC and SNOMED CT Interoperability Solution.
3. Lee, Preston Automated CBK Deployment Through Delivery Standards and the Skycapp Platform.
4. Mercer, Eric Making Model Checking Usable & Accessible for HIT Workflows.
5. Rashid, Sabbir A Demonstration of Computational Biomedical Knowledge Through the Performance of Ensemble Reasoning.
6. Seifi, Farid Demonstrating a Knowledge Object for CBK Containing Multiple Services and Implementations.
7. Shiell, Mitchell Orchestrating Genomics Data with Overture.

## OMOP‐2‐OPMI: ONTOLOGIZATION OF OMOP COMMON DATA MODEL (CDM) USING THE ONTOLOGY OF PRECISION MEDICINE AND INVESTIGATION (OPMI) FOR STANDARDIZED ELECTRONIC HEALTH DATA REPRESENTATION AND INTEGRATION

Saketh Boddapati, University of Michigan College of Literature, Science, and the Arts

bsaketh@umich.edu

Yongqun “Oliver” He, University of Michigan Medical School

yongqunh@med.umich.edu

Healthcare providers learn continuously as a core part of their work. However, as the rate of knowledge production in biomedicine increases, better support for providers' continuous learning is needed. Tools for learning from clinical data are widely available in the form of clinical quality dashboards and feedback reports. However, these tools seem to be frequently unused.

Making clinical data useful as feedback for learning appears to be a key challenge for health systems. Feedback can include coaching, evaluation, and appreciation, but systems developed for performance improvement do not adequately recognize these purposes in the context of provider learning. Moreover, providers have different information needs, motivational orientations, and workplace cultures, all of which affect the usefulness of data as feedback.

To increase the usefulness of data as feedback, we developed a Precision Feedback Knowledge Base (PFKB) for a precision feedback system. PFKB contains knowledge about how feedback influences motivation, to enable the precision feedback system to compute a motivational potential score for possible feedback messages. PFKB has four primary knowledge components: (1) causal pathway models, (2) message templates, (3) performance measures, and (4) annotations of motivating information in clinical data. We also developed vignettes about 7 diverse provider personas to illustrate how the precision feedback system uses PFKB in the context of anesthesia care. This ongoing research includes a pilot study that has demonstrated the technical feasibility of the precision feedback system, in preparation for a trial of precision feedback in an anesthesia quality improvement consortium.

## INTEROPERABILITY STANDARDS TO SUPPORT COMPUTABLE BIOMEDICAL KNOWLEDGE IN LEARNING HEALTH SYSTEMS

Bruce Bray, University of Utah, on behalf of the HL7 Learning Health Systems Work Group

bruce.bray@hsc.utah.edu

Data is the lifeblood of computable biomedical knowledge (CBK) and must adhere to standards to achieve the interoperability needed to generate virtuous learning cycles within a learning health system (LHS). The HL7 Learning Health System Work Group (HL7 LHS WG) conducted a scoping review to compile an initial list of standards that can support the LHS across “quadrants” of a virtuous learning cycle: (1) knowledge to action, (2) action to data, (3) data to evidence, and (4) evidence to knowledge. We found that few standards explicitly refer to an overarching framework that aligns interoperability and data standards across the phases of the LHS. We will describe our initial work to identify relevant gaps and overlaps in standards in this environment. Future work should address standards coordination and pilot testing within an LHS framework. These efforts can enhance collaboration among communities such as MCBK and HL7 to promote standards‐based computable knowledge mobilization.

## THE EVOLUTION OF THE KNOWLEDGE OBJECT MODEL ENHANCES THE FAIRNESS OF CBK

Allen Flynn, University of Michigan, on behalf of the Knowledge Systems Lab, Department of Learning Health Sciences

ajflynn@umich.edu

The Knowledge Object (KO) is a modular, extensible digital object, designed to allow computable biomedical knowledge (CBK) to be managed as a resource and implemented as a service. This poster describes how the KO model has evolved to better support the FAIR principles by:

Enabling multiple services to increase interoperability and reuse.

KOs were originally developed to run with an Activator, which loaded and deployed KOs on request, exposed the service, and routed responses as a RESTful API. While the intent was to facilitate low‐friction KO implementation, the Activator could limit interoperability and the potential for reuse. Expanding the model to reduce reliance on the Activator and allow for multiple services means KOs can meet a wider range of stakeholder needs. Current work includes developing updated specifications and reference implementation for activation.

Enabling multiple implementations of knowledge and services to increase interoperability and reuse. The legacy KO model included one implementation of both the CBK payload and the service to activate it. Engineering a KO to contain multiple implementations of CBK and services means KOs can meet a wider range of stakeholder needs. We are updating our model and engineering KOs capable of carrying multiple implementations and services.

Improving findability, accessibility interoperability, and reuse through an updated model and metadata. Metadata for the legacy KO model primarily described the service. Using the new model, we are now developing standards‐based extensible Linked Data metadata to describe the KO, the knowledge it contains, and the services that “activate” that knowledge.

## REAL‐WORLD MEASUREMENT EXPERIENCE TO MOBILIZE COMPUTABLE BIOMEDICAL KNOWLEDGE FOR PATIENT‐CENTERED CLINICAL DECISION SUPPORT

Nicole Gauthreaux, NORC at the University of Chicago

gauthreaux-nikki@norc.org

Courtney Zott, NORC at the University of Chicago

zott-courtney@norc.org

Prashila Dullabh, NORC at the University of Chicago

dullabh-prashila@norc.org

This poster is relevant to clinicians, health system leaders, informaticians, and researchers interested in driving future mobilization of computable knowledge for patient‐centered clinical decision support (PC CDS). We conducted a cross‐cutting synthesis of real‐world PC CDS projects to identify the types of measures used, measurement challenges and limitations, and action steps to advance PC CDS measurement. We reviewed research products from 20 PC CDS projects funded by the Agency for Healthcare Research and Quality (AHRQ) to gather information on their studies, and we conducted key informant interviews with Principal Investigators of nine projects to gather their experiences and challenges with PC CDS measurement.

Findings from the synthesis revealed a considerable focus on measuring the effectiveness of the PC CDS, primarily by collecting patient and clinician perspectives on the usability and acceptability of the tool and observing patient health outcomes from the intervention. Many projects incorporated patient perspectives in their study, yet there were more process measures (e.g., patient satisfaction with the design) than outcome measures (e.g., patient activation to manage their health due to the PC CDS). Few projects measured safety, or the technical performance and information presented by the PC CDS technology. Finally, equity measures rarely extended beyond descriptive analyses of participant socio‐demographics. Key informants described other evaluation challenges related to patient recruitment, technical limitations, and imprecision in data collection specific to PC CDS interventions. These findings provide a basis for guiding future development of measures that promote the adoption and use of knowledge for patient‐centered care.

## FROM INFORMATION TO INTELLIGENCE: DRIVING DATA TRANSFORMATION IN EMERGENCY MEDICINE

Pawan Goyal, American College of Emergency Physicians

pgoyal@acep.org

Data is driving the future of medicine. We've already seen the critical importance of real‐time insights to new and emerging health threats during the COVID‐19 pandemic, as well as the impact of health care trends and patterns of resource utilization. With the new Emergency Medicine Data Institute (EMDI), the American College of Emergency Physicians (ACEP) is rapidly moving emergency medicine to the forefront of data‐driven quality and practice innovation. This new initiative is poised to become a central source of intelligence and knowledge generation across all emergency medicine stakeholders. Harnessing the power of information that physicians are already recording, ACEP is synthesizing and standardizing data across multiple billing and EHR environments, innovating new research, and pursuing national‐level grants all while enhancing value for emergency physicians, patients, and the broader health care community.

## EXPLAINABLE AUTOMATED INCONSISTENCY DETECTION IN BIOMEDICAL AND HEALTH LITERATURE

Indika Kahanda, PhD, University of North Florida

n01530010@unf.edu

The quest for mobilizing computable biomedical knowledge, inconsistencies, and contradictions in biomedical literature would be a significant barrier. Given the exponential growth of scientific information, researchers often face the daunting task of detecting contradictory statements on crucial health topics. This work proposes to develop a full‐fledged, trustworthy automated pipeline for explainable contradiction detection, which will integrate an Information Retrieval (IR) system backed by a local data store, predictive models, and an Explainable AI (XAI) component. Users can input queries on medicine and health topics, and the system will identify top documents and sentences through syntactic analysis and refine results via semantic examination for relevant research claims. These sentences are forwarded to the predictive models backed by Large Language Models, which will classify each pair as contradictory. The XAI component will help output visual explanations based on these predictions. We have used ManConCorpus, a popular biomedical contradiction corpus on cardiovascular diseases, to develop and evaluate our predictive models. The preliminary results demonstrate that PubMedBERT, with an F1 score of 97%, can outperform BioBERT, Bioformer, and Distil‐BERT in classifying a given pair of sentences relevant to cardiovascular disease as contradictory or not. Further investigation is necessary to ensure that the models are robust in performing similarly on any health and medical topic. In the future, these predictive models will be combined with the aforementioned IR/XAI components for developing the prototype pipeline. This study has implications for medical and healthcare practitioners, researchers, students, systematic review authors, and the biomedical text‐mining community.

## A COMPUTABLE KNOWLEDGE BASE FOR PRECISION FEEDBACK

Zach Landis‐Lewis, University of Michigan, Department of Learning Health Sciences

zachll@umich.edu

Healthcare providers learn continuously as a core part of their work. However, as the rate of knowledge production in biomedicine increases, better support for providers' continuous learning is needed. Tools for learning from clinical data are widely available in the form of clinical quality dashboards and feedback reports. However, these tools seem to be frequently unused.

Making clinical data useful as feedback for learning appears to be a key challenge for health systems. Feedback can include coaching, evaluation, and appreciation, but systems developed for performance improvement do not adequately recognize these purposes in the context of provider learning. Moreover, providers have different information needs, motivational orientations, and workplace cultures, all of which affect the usefulness of data as feedback.

To increase the usefulness of data as feedback, we developed a Precision Feedback Knowledge Base (PFKB) for a precision feedback system. PFKB contains knowledge about how feedback influences motivation, to enable the precision feedback system to compute a motivational potential score for possible feedback messages. PFKB has four primary knowledge components: (1) causal pathway models, (2) message templates, (3) performance measures, and (4) annotations of motivating information in clinical data. We also developed vignettes about 7 diverse provider personas to illustrate how the precision feedback system uses PFKB in the context of anesthesia care. This ongoing research includes a pilot study that has demonstrated the technical feasibility of the precision feedback system, in preparation for a trial of precision feedback in an anesthesia quality improvement consortium.

## REPRESENTING AND INFERRING FROM MEDICAL KNOWLEDGE IN A TYPED DATABASE

Siddharth Limaye, Carle Illinois College of Medicine

slimaye2@illinois.edu

Understanding causation is an essential element of medical education. While a plethora of research exists regarding the use of knowledge graphs for topics such as drug discovery and personalized medicine, there is relatively less work regarding (1) the use of these graphs for medical education and (2) the use of causal graphs for these purposes. Pathology Graphs is a proof‐of‐concept application that uses a typed database to represent the causal model underlying several physiologic and pathophysiologic processes in the human body. Effectively, Pathology Graphs is an interactive, digital revision of the concept map.

While graph databases are suitable for building concept maps and causal graphs, Pathology Graphs instead uses a typed database to implement a higher‐order graph structure. This change allows Pathology Graphs to represent certain types of relationships (e.g., an enzyme modifying a reaction) which would otherwise not be simply expressible in a graph database. This change also allows inference of the downstream effects of a change in the pathology graph, such as predicting that product will decrease if an enzyme inhibitor is added to the system. This capability could be useful for further functionality, such as automatically generating multiple‐choice questions and answers for students or allowing students to generate differential diagnoses by examining all the potential causes of a finding. Challenges remaining to be addressed for this application include data input, user interface, and output visualization, as the goal of this project is to be accessible for those without pre‐existing programming knowledge.

## BRIDGING THE TRUST DIVIDE: HOW MEDICAL AI OUTPERFORMS YET BATTLES TO WIN PUBLIC CONFIDENCE—A STUDY CONDUCTED AMONG 74 UK RESIDENTS

Aswini Misro, YouDiagnose Limited

aswini@youdiagnose.online

Background—AI is being incorporated into healthcare by major tech companies, but public acceptance remains challenging. The study aims to understand resistance to AI despite its increasing accuracy and potential to improve patient waiting times.

Method—In partnership with the University of Hull and the Academic Health Science Network (AHSN) in the UK, a study was conducted that involved 111 adult patients or carers. 9 did not respond while 28 demonstrated poor digital literacy and were therefore excluded. An interactive user page was designed for the remaining users (*n* = 74) to engage with a medical chatbot. The participants were asked whether participants would use an AI‐powered medical chatbot or nurse for triage at their nearest A&E. Unstructured open‐ended interviews were conducted to understand the participants' reasoning behind their answers.

Finding – Participants ranged in age from 21 to 74, with a slight female majority (40 female vs. 34 male). The majority, 79.8% (*n* = 59) respondents, expressed their comfort with a nurse‐led care while a mere 20.2% (*n* = 15) showed readiness to interact with a medical chatbot. Notably, the age group of 20–40 was most open to the idea of consulting a chatbot. The four primary objections were: 90.5% (*n* = 67) believed that the chatbot fails to justify its decisions, 59.5% (*n* = 44) doubt its accuracy, 78.4% (*n* = 58) felt that the chatbot was inflexible, and 79.7% (*n* = 59) found it unemotional and detached.

Conclusion—Effective treatment relies on trust, respect, and understanding crucial elements needed when incorporating AI in medicine. It should be introduced progressively, considering patients' emotions and societal circumstances.

## IDENTIFYING CARE GAPS TO DRIVE CLINICAL QUALITY IMPROVEMENT: INTEROPERABLE, COMPUTABLE BIOMEDICAL KNOWLEDGE‐ENABLED APPROACHES ARE URGENTLY NEEDED

Jerome Osheroff, TMIT Consulting LLC/University of Utah/VA

josheroff@tmitconsulting.com

Dave Little (Epic) Stephanie Guarino (Nemours, ChristianaCare) Teresa Cullen (Pima County Health Dept) Rosie Bartel (Patient Partner) Joshua E. Richardson (RTI International) For the POLLC and SCD Learning Communities

Understanding gaps in care is essential for clinical quality improvement efforts. The Pain Management/Opioid Use LHS Learning Community (POLLC), an initiative that engages care delivery organizations (CDOs) and other stakeholders to collaboratively accelerate quality improvement efforts and results, has been working since 2022 to develop and implement a care gap report for this target. Participants identified 12 important potential care improvement opportunities to assess appropriate long‐term opioid use for chronic pain, for example, high opioid doses, multiple emergency department visits, and no Prescription Drug Monitoring Program Check. In March 2023, Epic released a pilot Care Gap Query that uses SQL code to identify patients meeting these criteria; it requires an Epic analyst with SQL expertise to implement and modify the query. In November 2023, Epic released a Care Gap Report (CGR) using a Reporting Workbench Template that enables Epic users without special expertise to run and configure the report. It enables users to take bulk action on report results, for example, placing orders and triggering communications. POLLC participants are exploring opportunities to aggregate care gap results from individual CDOs in a region into “population CGRs” that can be used to inform and guide public health interventions. A parallel learning community on sickle cell disease (SCD) that grew out of POLLC is developing analogous care gap reports for SCD in Epic and Oracle EHRs. Approaches are being explored to leverage interoperable, computable biomedical knowledge to make creating and deploying CGRs across targets, EHRs, and CDOs faster and more efficient.

## ELICITING SURVEY KNOWLEDGE WITH SEMANTIC DATA DICTIONARIES

Henrique Santos, Rensselaer Polytechnic Institute

oliveh@rpi.edu

Paulo Pinheiro, Instituto Piaget

pinhep@rpi.edu

Deborah L. McGuinness, Rensselaer Polytechnic Institute

dlm@cs.rpi.edu

Many countries perform surveys to gather data from their population for supporting decision‐making and development of public policies. Questionnaires are possibly the most used type of data acquisition instrument in surveys, although additional kinds may be employed (especially in health‐related surveys). In the United States, the NHANES is a national health and nutrition examination survey conducted by the National Center for Health Statistics, designed to collect data on adults and children's health and nutritional status. Data is organized in several tables, each containing variables to a specific theme, such as demographics, and dietary information. In addition, data dictionaries are available to (sometimes partially) document the tables' contents. While data is mostly provided by survey participants, instruments might be collecting data related to other entities (e.g., from participants' households and families, as well as laboratory results from participants' provided blood and urine samples). All this complex knowledge can often only be elicited by humans when analyzing and understanding the data dictionaries in combination with the data. The representation of this knowledge in a machine‐interpretable format could facilitate further use of the data. We detail how Semantic Data Dictionaries (SDDs) have been used to elicit knowledge about surveys, using the publicly available NHANES data and data dictionaries. In SDDs, we formalize the semantics of variables, including entities, attributes, and more, using terminology from relevant ontologies, and demonstrate how they are used in an automated process to generate a rich knowledge graph that enables downstream tasks in support of survey data analysis.

## OPEN EDUCATIONAL RESOURCES (OER) ON MCBK

Deborah Swain, North Carolina Central University

dswain@nccu.edu

Following MCBK pilot training in 2021–22 supported by an Institute of Museum and Library Services (IMLS) grant, we designed and developed an open educational resource (OER) platform to be accessible and sustainable for global users. Students and development partners included librarians in medical libraries and informatics graduate students in the United States and Canada. However, international applicants were unable to participate. The OER collection provides full access.

Subjects from the training in the OER website include:

- Overview of Mobilizing Computable Biomedical Knowledge manifesto and learning health systems cycle.
- Open access publishing introduction by editors, sample articles, and detailed instructions for submitting to the Learning Health Systems Journal.
- Community of Practice for health researchers, developers, and librarians covering systematic (PICO and PRISMA standards) and scoping reviews; concept of a metadata model for knowledge objects.
- Special topics—bias in AI and ML, guiding principles for MCBK technical infrastructure; entrepreneurial publishing, and trust and policy efforts for expanding MCBK.
- National Library of Medicine (NLM) roles related to computable knowledge.

The concept of an open pedagogy for resources supports open commons and sharing in the larger MCBK community. Policies for OER allow open content and open education practices. In the future, new material and potential courses and textbooks can become part of this MCBK OER collection of documents and slides.

Currently, the MCBK resources are hosted in the North Carolina Digital Online Collection of Knowledge (NC Docks): https://libres.uncg.edu/ir/nccu/clist.aspx?id=41690. Technical support is provided by UNC‐Greensboro and NCCU Research and Instructional Services Librarian, Danielle Colbert‐Lewis.

[See references and special contributors.]

## USING KM TO MANAGE AI BIAS IN HEALTHCARE

Deborah Swain, North Carolina Central University

dswain@nccu.edu

Amrit Vastrala, North Carolina Central University

avastral@eagles.nccu.edu

Bias in AI (artificial intelligence) and ML (machine learning) refers to the systematic errors that can occur in training data, algorithms, or models and lead to discriminatory or unfair outcomes for groups of people. This bias may be deliberate or inadvertent, and it may result from a number of factors including data collections, pre‐existing social biases, improper algorithm design, and ethical motivation of XAI (explainable AI).

The effects of bias can range from maintaining social injustices to making unreliable or inaccurate predictions to patients. The methodologies and frameworks for practice that biomedical researchers and health practitioners have proposed include fairness metrics, pre‐processing methods, post‐processing strategies to detect and mitigate bias, and models. Both model‐level explanations for providers and prediction‐level explanations for users/patients have been researched. This poster summarizes evidence‐based research and thoughtful recommendations for key stakeholders. Recognizing recent research and reference publications are the primary objective of our literature review and interviews.

Bias from AI is a difficult problem to solve due to complexity and nuance. There is still a lot of work to be done to ensure that biomedical systems are fair and transparent. Everyone involved can address bias in AI/ML and help develop best practices for building responsible and trustworthy systems. Ongoing research and collaboration among experts in ethics, social science, and healthcare will be essential.

## TOWARDS RECONSTRUCTION OF THE HUMAN PHENOTYPE ONTOLOGY PSYCHIATRY SECTION BY LEARNING FROM TRADITIONAL PSYCHIATRIC DIAGNOSTIC INFRASTRUCTURES

Yujia Tian, University of Michigan

yujiat@umich.edu

Although the etiology of most mental illnesses remains unclear, it is believed that they could be caused by a combination of genetic, social factors, and personal characteristics. The complex clinical manifestations of mental illnesses pose challenges in medical diagnosis. Many infrastructural models exist to support classification of mental illnesses. The Diagnostic and Statistical Manual of Mental Disorders, Fifth Edition (DSM‐5) serves as the principal authority for psychiatric diagnoses by the American Psychiatric Association (APA). The International Statistical Classification of Diseases and Related Health Problems (ICD) also provides a taxonomic and diagnostic classification of mental illnesses. However, DSM‐5 and ICD still struggle with accurate diagnosis with heterogeneity and comorbidity. In contrast, the Hierarchical Taxonomy Of Psychopathology (HiTOP) system offers a more continuous perspective of the disease spectrum. The Research Domain Criteria (RDoC) framework focuses on dimensions of behavioral/psychological functioning and their implementing neural circuits. Ontology brings a new direction to the future research of psychiatry. The Human Phenotype Ontology (HPO) standardizes various mental illnesses. HPO is also increasingly integrated with genomic data, enhancing its utility in clinical diagnostics and personalized medicine. We plan to integrate the advantages of existing infrastructure models in psychiatry into HPO and fill up missing aspects in the ontology. Ultimately, we can combine the concept of a learning health system (LHS) to create a medical environment that continuously learns and adapts, using big data and machine learning technology to optimize treatment strategies and improve the level of precision medicine.

## DEMONSTRATIONS INTEROPERABLE EHR DATA FOR COMPREHENSIVE CARE PLANNING, EVIDENCE GENERATION, AND IMPLEMENTATION AMONG PEOPLE LIVING WITH MULTIPLE CHRONIC CONDITIONS

Arlene Bierman, AHRQ

arlene.bierman@ahrq.hhs.gov

David Carlson, Clinical Cloud Solutions

dcarlson@clinicalcloud.solutions

Jenna Norton, NIDDK

jenna.norton@nih.gov

Evelyn Gallego, EMI Advisors

evelyn.gallego@emiadvisors.net

### Part 1

As the capacity for EHRs to share standardized data across providers and care settings, and to effectively present this information to providers and patients expands, the potential for generating and implementing computable biomedical knowledge to improve care delivery remains untapped. There are enormous gaps in the evidence on how to best provide care for the more than 25% of Americans, and 80% of those >65 who have multiple chronic conditions (MCC), as most research and practice guidelines are disease‐specific rather than person‐centered. The data and care of people living with MCC are fragmented, making data aggregation particularly important and challenging. The National Institute of Diabetes and Digestive and Kidney Diseases (NIDDK) and Agency Healthcare Research and Quality (AHRQ) partnered with multiple experts to develop open data standards and tools to support the documentation, exchange, and aggregation of electronic Care Plans (eCare) for people with MCC. To date, the project has developed and published data elements and value sets for chronic kidney disease (CKD) type 2 diabetes (T2D), cardiovascular disease (CVD), chronic pain, and long‐term COVID. These data elements and applicable use cases were incorporated into the HL7 Fast Health Interoperability Resource (FHIR) MCC eCare Plan Implementation Guide. The project developed two SMART on FHIR eCare plan applications (apps) based on the FHIR IG that can be used to integrate with the EHR to pull, share, and display key patient data to support care for people with MCC. The aim is to support comprehensive care planning across disparate systems of care and social services while keeping the person at the center of their care. By leveraging the 21st Century Cures Act requirement that patient data must be accessible by third‐party APIs, the eCare Plan (eCP) App V2 is able to request and receive USCDI‐outlined FHIR resources from any EHR system once the eCP app has been registered with that EHR's vendor. This aggregated data not only provides actionable information to plan care, enable shared decision‐making, identify, and achieve health goals, it improves the availability and quality of point‐of‐care data for use in pragmatic research. This project builds on existing resources developed by consensus‐based initiatives including the HL7 Argonaut and HL7 Gravity Project FHIR Accelerators. The goal is to promote the interoperable collection, use, and sharing of comprehensive, person‐centered health and social data across settings including goals, health‐related social needs, and patient‐reported outcomes. This presentation will demonstrate what standards and tools exist today to support interoperable care planning and how they can be used to generate computable biomedical knowledge to achieve evidence‐informed whole person care. The eCP also provides a platform for further development to incorporate clinical decision support.

### Part 2

The open source, HL7 balloted, SMART on FIHR, eCP promotes FAIR principles: Findability, Accessibility, Interoperability, and Reusability by enabling interoperability across EHRs, providing aggregated data to clinicians, patients, caregivers, and researchers for care planning, research, and quality improvement.

## THE LOINC ONTOLOGY: A LOINC AND SNOMED CT INTEROPERABILITY SOLUTION

Stanley Huff, MD, Graphite Health

stan.huff@graphitehealth.io

*Part 1*: I will be demonstrating a new LOINC Ontology. The LOINC Ontology is being made available as a SNOMED CT extension. The LOINC team at Regenstrief Institute and SNOMED International have a new agreement to make all LOINC content available in a new SNOMED CT extension. The creation of the new ontology is proceeding in a step‐wise fashion. The first content in the ontology is 24 000 quantitative laboratory tests. Information about the new ontology can be found at https://loincsnomed.org/ and a browser for the content can be found at https://browser.loincsnomed.org/.

*Part 2*: The new ontology allows sematic reasoning on LOINC content, a capability that has been lacking in previous releases of the LOINC terminology. For example, using the SNOMED Expression Constraint Language (ECL), you can easily identify the codes that represent fasting glucose levels regardless of the method used for the test. Future releases will allow deeper reasoning, for example, about what kinds of parenteral antibiotics are available for a particular kind of bacteria identified by culture.

## AUTOMATED CBK DEPLOYMENT THROUGH DELIVERY STANDARDS AND THE SKYCAPP PLATFORM

Preston Lee, PhD, MBA, FAMIA – Skycapp

preston.lee@prestonlee.com

Adela Grando, PhD, FACMI, FAMIA – Arizona State University

adela.grando@asu.edu

*Part 1*: This Skycapp software delivery platform demo will show how a complex NIH‐funded CDS Hooks service developed at Arizona State University (ASU) can be widely disseminated and deployed in an automated manner to worldwide evaluators and adopters. The system and approach are generalized to all “level 4” CBK types through strict adherence to FHIR (and other) data standards and infrastructural interoperability.

*Part 2*: Skycapp's implementation of post‐publication CBK delivery and deployment is based on the balloted HL7/Logica Marketplace 2 STU 2 specification. We have purposefully designed the platform to provide a “publish → deploy → adopt” model of dissemination enabling adopters to evaluate artifacts in local context prior to deciding to pursue their adoption, thus encouraging CDS experimentation by deferring any sizable commitments of time or money.

## MAKING MODEL CHECKING USABLE & ACCESSIBLE FOR HIT WORKFLOWS

Eric Mercer, Brigham Young University, Computer Science

eric.mercer@byu.edu

Bryce Pierson, Brigham Young University, Computer Science

bryce.mckay.pierson@gmail.com

Keith A. Butler, University of Washington

keith.a.butler@gmail.com

*Part‐1*: The CBK of our live demo will cover:

- A complex HIT workflow for remote patient monitoring (RPM), modeled in BPMN [1] that distributes cognition across patients at home with the app on smart phones, AI in the cloud, and providers in their offices with their app on browsers;
- Automatic translation from the BPMN graphical workflow into Promela [2], a formal process modeling language.
- The evaluation criterion for model checking [3] the RPM design: a technology‐independent, finite‐state machine for the cognitive work problem the RPM workflow must solve: “Maintain actionable risk awareness of the patient's condition”[4].
- The correctness analysis of the workflow's design by the SPIN model‐checker [2] and the meaning of this workflow's verification certificate output by SPIN.
- Automatic translation of any design flaws reported by SPIN and mapped back to a BPMN model for fault isolation.

We will conclude by summarizing expected benefits for

- patient safety from thorough fault discovery before HIT gets too expensive to change.
- decreased provider burden of HIT use;
- medical personnel to read and critique BPMN's graphical workflows and participate in design revisions.

*Part‐2*: FAIR Principles Relevance

Findable: Complex HIT designs could be indexed for search engine discovery by the cognitive work problem they were proven and certified to solve.

Accessible: Our overall aim is automated translation back and forth between the concepts and languages of the design community and those of model checking, thereby making model checking far more accessible and usable for provider participation in HIT design.

Interoperable: The BPMN standard [1] is widely adopted and available in dozens of commercially supported modeling products. BPMN models can be exported as XML files, thereby carrying forward the conceptual design requirement onto implementation platforms.

Reusable: The finite state machine for the cognitive work problem that a workflow design must solve can be reused for model checking multiple HIT designs that purport to solve the same cognitive problem. Certification means they each can solve the identical problem, yet may differ widely in their qualities of usability, function allocation to human vs. computing, cost to develop/deploy, timeliness, etc.

The important, related principle of trustworthiness is also increased by model checking certificates that verify all sequences of an HIT design are correct.

Notes:

1. Business Process Modeling + Health. An OMG project adapting the Business Process Modeling Notation (BPMN) to health care workflows at https://www.bpm-plus.org/index.htm
2. Holzmann, G., Najm, E., & Serhrouchni, A. (2000). SPIN model checking: An introduction. International Journal on Software Tools for Technology Transfer, 2, 321–327.
3. ACM 2007 Turing Award for Model Checking. https://amturing.acm.org/award_winners/clarke_1167964.cfm
4. This cognitive work problem was adapted from: NIH Therapeutic Management of Nonhospitalized Adults with COVID‐19, accessed at: https://adsp.nm.org/uploads/1/4/3/0/143064172/nih_non-hosp_adult_guideline.pdf

## A DEMONSTRATION OF COMPUTATIONAL BIOMEDICAL KNOWLEDGE THROUGH THE PERFORMANCE OF ENSEMBLE REASONING

Sabbir Rashid, Rensselaer Polytechnic Institute

rashis3@rpi.edu

Deborah McGuinness, Rensselaer Polytechnic Institute

dlm@cs.rpi.edu

A clinical decision support system (CDSS) can support physicians in making clinical decisions, such as differential diagnosis, therapy planning, or plan critiquing. To make such informed decisions, there may be a large amount of medical data and literature that a physician needs to keep track of such as new research articles, pharmacological therapies, and updates in Clinical Practice Guidelines. Therefore, a CDSS can be designed to assist physicians by providing relevant, evidence‐based clinical recommendations, subsequently reducing the mental overhead required to keep up to date with an evolving body of literature. We designed a CDSS by leveraging Semantic Web technologies to create an AI system that reasons in a way similar to physicians. We base our abstraction of human reasoning on the Select and Test Model (ST‐Model), which combines multiple forms of reasoning, such as abstraction, deduction, abduction, and induction, to arrive at and test hypotheses. Based on this framework, we perform ensemble reasoning, the integration and interaction of multiple types of reasoning. We apply our CDSS to the treatment of type 2 diabetes mellitus by designing a domain ontology, the Diabetes Pharmacology Ontology (DPO), that supports both deductive and abductive reasoning. DPO is additionally used to provide a schema for our knowledge representation of hypothetical patients, where each patient is encoded in RDF as a Personalized Health Knowledge Graph (PHKG). We build our system using the Whyis knowledge graph framework by writing software agents to perform custom deductive reasoning and integrate the performance of abduction using an existing reasoning engine, the AAA Abduction Solver. We apply our approach to perform therapy planning on the hypothetical patients, which we will showcase as a part of the demonstration of our system.

The use of semantic technologies allows us to leverage existing reasoning engines, both in terms of deductive and abductive reasoning, use formal knowledge representations, such as clinical ontologies and vocabularies, and incorporate existing techniques for capturing provenance, such as nanopublications. Additionally, our approach allows for the generation of justifications for the reasoning choices made. Furthermore, this work promotes the FAIR principles and guidelines that have been widely adopted and cited for publishing data and metadata on the web. For the ontology to be findable, globally unique and persistent identifiers are created for each resource in the ontology. Concepts are directly accessible from their URL and the ontology itself is directly accessible via the resource URL defined in the ontology. To promote interoperability, we link concepts in our ontology to other standard vocabularies, including LOINC, ChEBI, Symptom Ontology, and NCIT. Finally, to promote the reusability of our resource, we have published, made readily available, and adequately documented the ontology, PHKGs, and software that we use for our CDSS. The demonstration will show our hybrid reasoning clinical decision support system in action in a diabetes setting.

## DEMONSTRATING A KNOWLEDGE OBJECT FOR COMPUTABLE BIOMEDICAL KNOWLEDGE CONTAINING MULTIPLE SERVICES AND IMPLEMENTATIONS

Farid Seifi, Knowledge Systems Lab, University of Michigan

faridsei@med.umich.edu

Anurag Bangera, Knowledge Systems Lab, University of Michigan

abangera@umich.edu

The Knowledge Object (KO) is a modular, extensible container for computable knowledge. Since 2018, we have developed over 100 KOs for computable biomedical knowledge (CBK). Through this experience, we identified limitations in the legacy KO model, leading to further development of the conceptual model. This new model better supports the FAIR principles, particularly interoperability and reusability. In this technical demonstration, we contrast a legacy KO with the new KO model to illustrate the capabilities for reuse and interoperability enabled by the new model.

1. The CBK to be Demonstrated

The KO model is domain‐agnostic and can be applied across many different types of computable knowledge. The examples used in this demo will include personalized models for preventive medicine, but the focus is on the metamodel rather than a specific instance of CBK. The demo will showcase developments to two specific aspects of the KO model:

- Service: a way to access computable knowledge contained in a KO, e.g., via a web API, command line interface, and as a stand‐alone web application.
- Implementation: a representation of the computable knowledge or service in a single programming language.

Additionally, we have improved on our original metadata model and will discuss how standards‐based Linked Data metadata improves the Findability, Accessibility, Interoperability, and Reusability of CBK packaged as KOs.

Legacy KOs could only be run as part of a RESTful web service that can be called by other systems. Additionally, the legacy KO model allowed for only one service and implementation. In contrast, the enhanced model allows for multiple services within a single KO, and multiple implementations of the same knowledge and/or services, each in a different programming language. In this demo, we will show how the files, metadata, code, and other information needed to a variety of technical paths can be packaged together inside a single compound digital Knowledge Object that has the potential to support reuse by a variety of people with different professional roles.

The enhanced KO model creates new possibilities to access and implement knowledge and services. Rather than being limited to one service, the new model enables the following capabilities:

- Run as a stand‐alone API application
- Facilitate access to the CBK inside a KO through CLI (by applications or users);
- Allow developers to use the knowledge and services directly inside of code as a library, in a native way, (e.g., directly embedded into a larger software program as a function).

2 Promoting Interoperability and Reusability, the I,R in FAIR

The enhanced KO model can package computable biomedical knowledge in a technically variform way so that a wider variety of stakeholders can more quickly and easily use it. By offering many technical paths to deploying and using the same computable knowledge, CBK artifacts can be used in multiple different contexts, increasing interoperability and reusability. Enabling various technical paths to using the same CBK provides different ways for application developers, system integrators, CBK evaluators, data analysts, and others to reuse CBK in ways that meet existing and emerging needs.

## ORCHESTRATING GENOMICS DATA WITH OVERTURE

Mitchell Shiell, Ontario Institute for Cancer Research

mshiell@oicr.on.ca

Describe the computable biomedical knowledge (CBK) you will demonstrate:

Next‐generation sequencing has made genomics datasets commonplace, posing new challenges for research groups who want to efficiently gather, store, and share their data, while maximizing its value and reuse. This creates a compelling case for new computational tools to mobilize these massive datasets at scale. Overture is a suite of open‐source and free‐to‐use modular software that works in concert to build and deploy scalable genomics data platforms. These platforms streamline the gathering, organizing, and sharing of raw and interpreted data, making it accessible for both humans and machines to translate into knowledge.

Our MCBK demo will highlight how Overture creates data resources that broadly achieve FAIR data goals. We will demonstrate how our core microservices—Ego, Song, Score, Maestro, and Arranger—achieve these data goals with a presentation and practical demonstration of the Overture platform.

Describe how your CBK promotes the FAIR principles and/or trust:

Overture is comprised of five core components that each provide a foundation for mobilizing discoverable, FAIR (Findable, Accessible, Interoperable, and Reusable) genomics data. (1) Ego, Overture's identity and permission management service, enables accessibility with appropriate authentication and authorization procedures using standard and free protocols. (2) Song and (3) Score work together to support findability with data submission, management, and retrieval methods. These services significantly increase data quality, findability, and interoperability with automated tracking and custom metadata validations. (4) Maestro indexes data from a distributed network of Song metadata repositories into a unified Elasticsearch index, and (5) Arranger then uses this index to produce a graphQL search API that can be extended with a library of configurable search and portal UI components. Combining these services completes a comprehensive end‐to‐end data portal that broadly enables the secure, scalable reuse of genomics data. Overture aims to make large‐scale genomics data FAIR and cost‐effective for researchers worldwide, fostering mobilization and collaboration over data globally.

